# Motor activity in gamma and high gamma bands recorded with a Stentrode from the human motor cortex in two people with ALS

**DOI:** 10.1088/1741-2552/adbd78

**Published:** 2025-03-31

**Authors:** Kriti Kacker, Nikole Chetty, Ariel K Feldman, James Bennett, Peter E Yoo, Adam Fry, David Lacomis, Noam Y Harel, Raul G Nogueira, Shahram Majidi, Nicholas L Opie, Jennifer L Collinger, Thomas J Oxley, David F Putrino, Douglas J Weber

**Affiliations:** 1 Department of Mechanical Engineering, Carnegie Mellon University, Pittsburgh, PA, United States of America; 2 NeuroMechatronics Lab, Carnegie Mellon University, Pittsburgh, PA, United States of America; 3 The Neuroscience Institute, Carnegie Mellon University, Pittsburgh, PA, United States of America; 4 Center for Neural Basis of Cognition, Pittsburgh, PA, United States of America; 5 Vascular Bionics Laboratory, Department of Medicine, Royal Melbourne Hospital, University of Melbourne, Parkville, Victoria, Australia; 6 Synchron Inc., New York, NY, United States of America; 7 Departments of Neurology and Pathology (Neuropathology), University of Pittsburgh School of Medicine, Pittsburgh, PA, United States of America; 8 James J. Peters VA Medical Center, Bronx, NY, United States of America; 9 Department of Rehabilitation and Human Performance, Icahn School of Medicine at Mount Sinai, New York, NY, United States of America; 10 Department of Neurology and Neurosurgery, University of Pittsburgh Medical Center, Stroke Institute, Pittsburgh, PA, United States of America; 11 Department of Neurosurgery, Icahn School of Medicine at Mount Sinai, New York, NY, United States of America; 12 Rehab Neural Engineering Labs, University of Pittsburgh, Pittsburgh, PA, United States of America; 13 Department of Physical Medicine and Rehabilitation, University of Pittsburgh, Pittsburgh, PA, United States of America; 14 Department of Bioengineering, University of Pittsburgh, Pittsburgh, PA, United States of America; 15 Department of Biomedical Engineering, Carnegie Mellon University, Pittsburgh, PA, United States of America

**Keywords:** brain–computer interface, Stentrode, ALS, paralysis, clinical trial, electrocorticography, BCI

## Abstract

*Objective.* This study examined the strength and stability of motor signals in low gamma and high gamma bands of vascular electrocorticograms (vECoG) recorded with endovascular stent-electrode arrays (Stentrodes) implanted in the superior sagittal sinus of two participants with severe paralysis due to amyotrophic lateral sclerosis. *Approach.* vECoG signals were recorded from two participants in the COMMAND trial, an Early Feasibility Study of the Stentrode brain–computer interface (BCI) (NCT05035823). The participants performed attempted movements of their ankles or hands. The signals were band-pass filtered to isolate low gamma (30–70 Hz) and high gamma (70–200 Hz) components. The strength of vECoG motor activity was measured as signal-to-noise ratio (SNR) and the percentage change in signal amplitude between the rest and attempted movement epochs, which we termed depth of modulation (DoM). We trained and tested classifiers to evaluate the accuracy and stability of detecting motor intent. *Main results.* Both low gamma and high gamma were modulated during attempted movements. For Participant 1, the average DoM across channels and sessions was 125.41 ± 17.53% for low gamma and 54.23 ± 4.52% for high gamma, with corresponding SNR values of 6.75 ± 0.37 dB and 3.69 ± 0.28 dB. For Participant 2, the average DoM was 22.77 ± 4.09% for low gamma and 22.53 ± 2.04% for high gamma, with corresponding SNR values of 1.72 ± 0.25 dB and 1.73 ± 0.13 dB. vECoG amplitudes remained significantly different between rest and move periods over the 3 month testing period, with >90% accuracy in discriminating attempted movement from rest epochs for both participants. For Participant 1, the average DoM was strongest during attempted movements of both ankles, while for Participant 2, the DoM was greatest for attempted movement of the right hand. The overall classification accuracy was 91.43% for Participant 1 and 70.37% for Participant 2 in offline decoding of multiple attempted movements and rest conditions. *Significance.* By eliminating the need for open brain surgery, the Stentrode offers a promising BCI alternative, potentially enhancing access to BCIs for individuals with severe motor impairments. This study provides preliminary evidence that the Stentrode can detect discriminable signals indicating motor intent, with motor signal modulation observed over the 3 month testing period reported here.

## 1. Introduction

Brain–computer interfaces (BCIs) provide a means to communicate and control assistive devices for people with severe paralysis by recording and decoding motor signals to discern user intent (Wolpaw 2007). For example, electrodes placed on or within the motor cortex can measure electrical signals generated by neurons engaged in motor planning and execution (Schalk and Leuthardt 2011). The properties of these motor signals vary with sensor location. Electrodes for BCIs are placed on the scalp or implanted surgically, typically targeting the motor cortex. Scalp electrodes are used for electroencephalography (EEG), which poses virtually no risk of injury and has shown promising results for BCI control of assistive technologies (Cincotti *et al* 2008, Sellers *et al* 2010). Since the bandwidth of scalp EEG is limited, the frequency ranges generally chosen to detect motor-related cortical activity are alpha (8–13 Hz) and beta (13–30 Hz) (Comani *et al* 2015, Orban *et al* 2022). Motor preparation and action are associated with desynchronization in the alpha and beta bands, which serves as a marker of motor intent that can be detected for BCI applications (Pfurtscheller *et al* 1997, Crone 1998). However, EEG is susceptible to noise, motion, and ocular artifacts, and requires assistance for daily setup (Schwartz *et al* 2006, Yuan and He 2014, Opie *et al* 2016, Orban *et al* 2022). EEG also provides less information than intracortical or subdural signals due to the temporal and spatial filtering caused by intervening tissues and cerebrospinal fluid (Slutzky *et al* 2010). Furthermore, the effectiveness of scalp EEG in capturing task-specific signals can be limited by the distance between the scalp and cortex (Saha *et al* 2021).

To improve signal quality, electrode arrays can be placed on the brain surface for electrocorticography (ECoG), to measure field potentials (Leuthardt *et al* 2004). However, implantation of these devices necessitates invasive surgery to remove a portion of the skull to access the brain. Consequently, there are risks associated with infection and bleeding (Rolston *et al* 2016, Branco *et al* 2023, Ji *et al* 2023). Traditionally, ECoG grids have been used diagnostically to localize epileptogenic zones (Kuruvilla and Flink 2003). Recently, researchers have explored ECoG-based BCI in people with paralysis (Moses *et al* 2021, Metzger *et al* 2023). ECoG-based BCI analysis primarily focuses on low gamma and high gamma frequency bands, which provide more localized detection of neural activity and more action-specific control signals for BCI in comparison to alpha and beta frequency bands (Schalk and Leuthardt 2011, Chestek *et al* 2013, Blakely *et al* 2014, Branco *et al* 2017, Freudenburg *et al* 2019). ECoG BCI studies in participants with tetraplegia have demonstrated robust multi-degree cursor movements using high-density ECoG grids placed on the motor cortex (Wang *et al* 2013).

To access information contained within an even smaller population of neurons, intracortical microelectrodes such as Neuroport electrode (Blackrock Neurotech, Salt Lake City, UT, USA) penetrate the brain surface and enable detection of action potentials from isolated and clusters of neurons (Schwartz *et al* 2006). By recording from populations of neurons in the motor cortex, it is possible to decode complex, high-dimensional motor actions such as reaching and grasping, and even speech (Hochberg *et al* 2012, Collinger *et al* 2013, Willett *et al* 2023). However, in addition to requiring a craniotomy to access the brain, these electrode arrays are also penetrated directly through and into delicate neural tissue, carrying an additional risk of device rejection and cerebral trauma (McConnell *et al* 2009, Wang *et al* 2023). Prior literature on deep brain stimulation implants and intracortical arrays has reported infection rates around 2%–5% and hemorrhage around 3% (Bullard *et al* 2020). Moreover, the most common device-related adverse event in the BrainGate2 trial was skin irritation around the percutaneous pedestal (Rubin *et al* 2023), which is not a problem with fully implanted systems.

These examples demonstrate the breadth of BCI technologies and the variety of neural signal features that are being developed. A relatively new addition to the lineup of BCI technologies is a device called the Stentrode (Synchron, Inc., Brooklyn, New York, USA). The Stentrode is an intracranial electrode delivered endovascularly, eliminating the need for craniotomy. Positioning the Stentrode in the superior sagittal sinus (SSS) enables direct access to the dorsal regions of the motor cortex (Martini *et al* 2020). The SWITCH trial was the first-in-human study of the Stentrode, which took place in Australia and demonstrated stable signal bandwidth over 12 months in four participants (Oxley *et al* 2021), paving the way for the COMMAND trial, an Early Feasibility Study (EFS) currently underway in the United States (NCT05035823). The results of the SWITCH study report no vessel occlusion or device migration (Mitchell *et al* 2023).

Prior to implantation in humans, the Stentrode underwent extensive testing in sheep models. This included evaluation of spatial resolution, signal-to-noise ratio (SNR), and bandwidth which has been reported as comparable to epidural ECoG arrays (Oxley *et al* 2016, John *et al* 2018). Meanwhile, the findings from the SWITCH study primarily address the safety of the Stentrode in humans and its potential for enabling device control (Oxley *et al* 2021, Mitchell *et al* 2023). To date, the SNR and other properties of motor signals recorded by the Stentrode in clinical trials have not been reported. Similar to other implanted methods, the Stentrode is still susceptible to some artifacts, such as breathing artifacts (Mitchell *et al* 2023), though local referencing techniques are used to mitigate non-neural interference.

This report provides a preliminary examination of motor signals recorded by the Stentrode from two subjects enrolled in the COMMAND EFS. We present preliminary results on the amplitude, signal quality, and stability of low gamma and high gamma signals associated with attempted movement in two participants with severe paralysis due to amyotrophic lateral sclerosis (ALS) enrolled in the United States trial. Participants in this study performed a variety of tasks. For this paper, we focused only on datasets for motor mapping studies that were performed consistently over a three month span. For Participant 1, the data spans the 4th, 5th, and 6th months after implantation, while for Participant 2, it covers the 8th, 9th, and 10th months. We examined motor signal strength over time and compared the modulation of signals associated with attempted movements of different limbs.

We demonstrated that signals recorded by the Stentrode show significant amplitude modulation in the low gamma and high gamma frequency bands during attempted movement. The amplitude differences between rest and attempted movements remained significant over a 3 month period. The attempted movement events were detectable with over 90% accuracy using a single subject-specific classifier over the 3 month interval. Additionally, we found that the motor signals generated during attempted movements of different limbs were separable, which may eventually make it possible to control multiple degrees of freedom.

## 2. Methods

### 2.1. Participants

Two participants (P1 and P2) with severe full-body paralysis due to advanced ALS were implanted with the Stentrode as part of the ongoing COMMAND EFS trial (NCT05035823). These participants are the first two to be implanted with the Stentrode from the COMMAND EFS trial. This study was conducted under an Investigational Device Exemption from the U.S. Food and Drug Administration and approved by the Institutional Review Boards at Western-Copernicus Group, Mount Sinai Hospital, University of Pittsburgh, University of Buffalo, and Carnegie Mellon University. Informed consent was obtained before any study procedures were conducted.

Both participants were male (P1: 67 years old and P2: 73 years old at the time of implantation) with advanced ALS resulting in complete paralysis of their extremities and severely diminished respiratory function. They were ventilator-dependent and unable to speak, but could use an eye tracker for communication and computer access.

### 2.2. Device details

**Figure 1.**
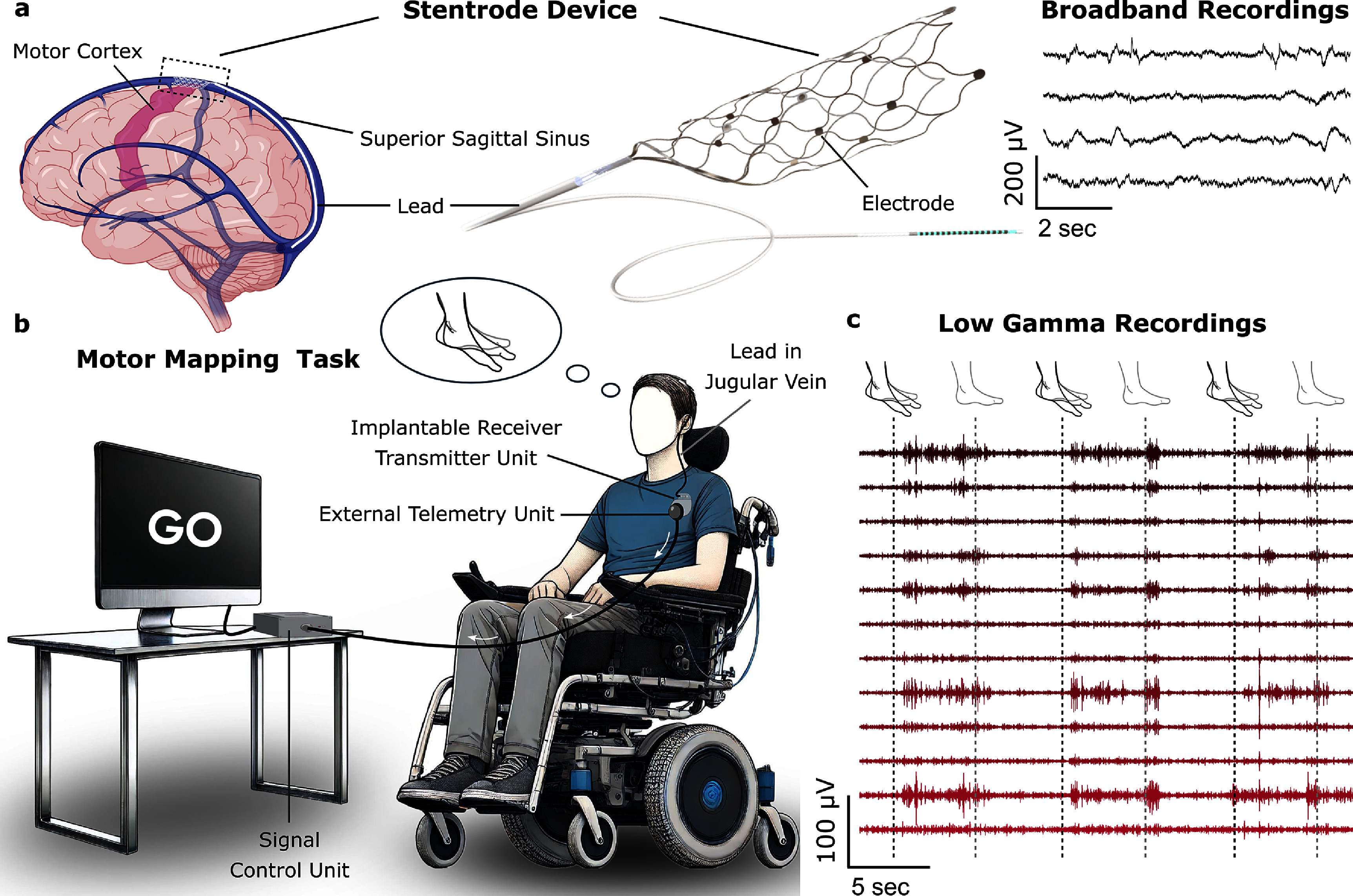
Schematic representation of the fully implanted Stentrode brain–computer interface and examples of signals recorded with the device. (a) The Stentrode is implanted in the superior sagittal sinus and sits adjacent to the primary motor cortex of both hemispheres. The Stentrode is a 16 channel flexible electrode array designed to expand and conform to the walls of the superior sagittal sinus adjacent to the primary motor cortex. Neural data is recorded from the Stentrode as participants attempt limb movements. (b) Diagram of the experimental setup while the participant performs the motor mapping task. The Stentrode’s lead connects to an implantable receiver-transmitter unit housed in a subcutaneous pocket, which wirelessly transmits neural signals to an external telemetry unit connected to a host computer. Participants are instructed to attempt limb movements based on visual cues displayed on a screen. (c) Participants are presented with alternating cues for attempted movement and rest during data collection. The figure illustrates sample low gamma band-pass filtered recordings from 12 channels, recorded while Participant 1 attempted bilateral ankle movements in response to cues.

The Stentrode consists of 16 platinum electrodes, each with a 500 *μ*m diameter, mounted on a self-expanding nitinol scaffold measuring 8 × 40 mm (Stentrode, Synchron, CA, USA) (figure 1(a)) (Oxley *et al* 2021). The interelectrode spacing is approximately 3 mm (Mitchell *et al* 2023).

Prior to implantation, structural and functional magnetic resonance imaging (fMRI) was performed to localize and verify activation in the primary motor cortex (M1) in relation to the SSS. Participants were prompted to attempt flexion and extension movements of one or both ankles during fMRI scanning. The Stentrode is inserted through the jugular vein into the SSS and positioned over the M1 between both hemispheres. Once deployed within the vasculature, the Stentrode expands and conforms to the walls of the SSS adjacent to M1. Immediately after device deployment, contrast-enhanced 3D digital subtraction angiography showed full opening of the stent-electrodes and secure apposition of the device against the wall of the SSS. The electrodes are connected via a flexible lead to an implantable receiver transmitter unit (IRTU, Synchron, NY, USA) placed subcutaneously in the chest below the clavicle. One channel on the Stentrode was designated as a common reference electrode for the remaining electrodes. The signals were sampled at a rate of 2000 Hz and transmitted wirelessly to an external telemetry unit (figure 1(b)). A detailed description of the device, surgical methodology, and signal acquisition has been described previously (Oxley *et al* 2021, Mitchell *et al* 2023). The impedance of all electrode channels was measured during each session using a test current of 10 nA at 100 Hz. The average impedance and standard deviation across sessions for all channels included in this study’s analysis are reported (tables 1 and 2).

**Table 1.** Mean and standard deviation (SD) of depth of modulation and signal-to-noise ratio (low and high gamma bands) values for channels across 64 runs spanning 8 sessions for Participant 1. The reference channel had an average impedance of 38 (1.3) kOhms across these 8 sessions. Bold values indicate the mean (SD) of impedance, depth of modulation, and signal-to-noise ratio averaged across all channels.

Channel number	Impedance (kΩ)	Depth of modulation		Signal-to-noise ratio	
		Low gamma (%)	High gamma (%)	Low gamma (dB)	High gamma (dB)
Channel 1	104 (10.2)	220.79 (66.81)	63.13 (14.07)	9.96 (1.68)	4.22 (0.76)
Channel 2	19 (0.5)	118.73 (34.69)	60.11 (5.55)	6.69 (1.4)	4.08 (0.3)
Channel 3	24 (1.0)	101.4 (27.93)	49.87 (8.59)	5.99 (1.26)	3.50 (0.49)
Channel 4	29 (0.9)	113.55 (35.32)	52.71 (10.21)	6.48 (1.33)	3.66 (0.59)
Channel 5	31 (1.2)	113.35 (36.79)	51.16 (13.95)	6.46 (1.43)	3.55 (0.84)
Channel 6	34 (1.6)	83.52 (17.87)	45.62 (14.6)	5.24 (0.8)	3.22 (0.87)
Channel 7	8 (13.4)	107.51 (28.24)	67.16 (12.18)	6.26 (1.18)	4.44 (0.66)
Channel 8	42 (0.6)	197.36 (54.3)	61.12 (16.31)	9.32 (1.62)	4.10 (0.91)
Channel 9	41 (1.6)	91.01 (24.82)	43.15 (8.01)	5.55 (1.1)	3.10 (0.5)
Channel 10	25 (0.3)	93.27 (24.83)	50.1 (16.16)	5.65 (1.17)	3.48 (0.96)
Channel 11	41 (1.7)	179.51 (101.02)	63.87 (43.24)	8.3 (3.49)	3.96 (2.48)
Channel 12	68 (8.5)	84.98 (50.28)	42.76 (23.91)	5.06 (2.15)	2.97 (1.48)
**Mean (SD)**	**39 (3.3)**	**125.41 (17.53)**	**54.23 (4.52)**	**6.75 (0.37)**	**3.69 (0.28)**

**Table 2.** Mean and standard deviation of depth of modulation and signal-to-noise ratio (low and high gamma bands) values for channels across 110 runs spanning 11 sessions for Participant 2. The reference channel had an average impedance of 42 (1.9) kOhms across these 11 sessions. Bold values indicate the mean (SD) of impedance, depth of modulation, and signal-to-noise ratio averaged across all channels.

Channel number	Impedance (kΩ)	Depth of modulation		Signal-to-noise ratio	
		Low gamma (%)	High gamma (%)	Low gamma (dB)	High gamma (dB)
Channel 1	36 (0.7)	18.39 (13.99)	19.27 (8.11)	1.41 (1.02)	1.51 (0.60)
Channel 2	28 (0.7)	25.53 (11.94)	25.52 (10.22)	1.94 (0.81)	1.94 (0.72)
Channel 3	29 (2.6)	19.74 (6.89)	21.89 (8.78)	1.55 (0.49)	1.70 (0.64)
Channel 4	21 (0.4)	25.21 (14.98)	23.91 (8.12)	1.89 (1.03)	1.84 (0.58)
Channel 5	3 (0.2)	35.98 (20.2)	31.51 (12.1)	2.57 (1.29)	2.34 (0.83)
Channel 6	28 (0.8)	20.61 (13.56)	20.07 (7.68)	1.57 (0.96)	1.57 (0.54)
Channel 7	32 (0.6)	23.93 (11.62)	24.67 (8.84)	1.83 (0.79)	1.89 (0.65)
Channel 8	54 (0.9)	12.81 (8.65)	13.38 (7.46)	1.02 (0.66)	1.07 (0.58)
**Mean (SD)**	**30 (1.0)**	**22.77 (4.09)**	**22.53 (2.04)**	**1.72 (0.25)**	**1.73 (0.13)**

### 2.3. Motor signal testing

In general, participants completed two testing sessions per week, conducted in their homes, with each session lasting up to four hours. Motor mapping experiments involved attempted movements of various body parts. Testing typically began in the first testing session, though the number and types of tests varied over time and among participants. For this study we selected a subset of data over a 3 month period involving motor mapping with a consistent limb movement for each participant. None of the tests, which had consistent conditions, were excluded from the analysis.

Each session included trials with 8–10 alternating *rest* and *go* phases, ensuring consistent intervals within each trial. During the *go* periods, participants were instructed to attempt moving either their ankles or hands. During the *rest* periods, participants were instructed to relax and not think about anything in particular. When cued to attempt hand movements, participants were instructed to try opening and closing their fists. For cues related to ankle movements, participants were instructed to attempt flexing and extending their ankles. Participants did not practice these movements outside the experimental sessions.

For P1, the duration of the rest period (4.98 ± 0.56 s) varied to prevent the participant from anticipating the *go* cue to start the attempted movement. The participant was instructed to attempt five repetitions of the movement during the *go* cue period, which was 5 s in duration. For P2, each trial included a 10 s go window following a 10 s rest period, with the trial advancing when the subject generated the desired output. For the analysis in this paper, we focused on the data from go and rest periods. P2 was instructed to attempt one brisk movement during the go cue window. 12 channels were used for P1 and 8 channels were used for P2.

The type of attempted movement was chosen from one of the following six options: right hand, left hand, both hands, right ankle, left ankle, and both ankles. In this study, the primary analysis focuses on the attempted movement of both ankles for P1 and the attempted movement of the right hand for P2. We quantified changes in the vascular electrocorticograms (vECoG) signals between rest and attempted movement over several months for both participants, focusing on these actions. Additionally, we used a variety of attempted movement tasks to compare motor activity patterns across the Stentrode during different movements. To ensure consistency in training the linear discriminant analysis (LDA) classifier for multiple limb movements, we pooled data from three sessions, selecting days that were as close together as possible. For P1, we compared signals for attempted movement of the right ankle, left ankle, and both ankles. Data was collected on days 137, 148, and 157 post-implantation. For P2, we compared signals for attempted movement of the right hand, left hand, and both hands. Data was collected on day 97 post-implantation.

### 2.4. Data analysis

All analyses were conducted using custom Python code. A 4th-order Butterworth high-pass filter with a cut-off frequency of 0.5 Hz was applied to remove low-frequency noise and a 2nd-order infinite impulse response notch filter at 60 Hz was used to eliminate line noise.

#### 2.4.1. Power spectral density (PSD)

**Figure 2.**
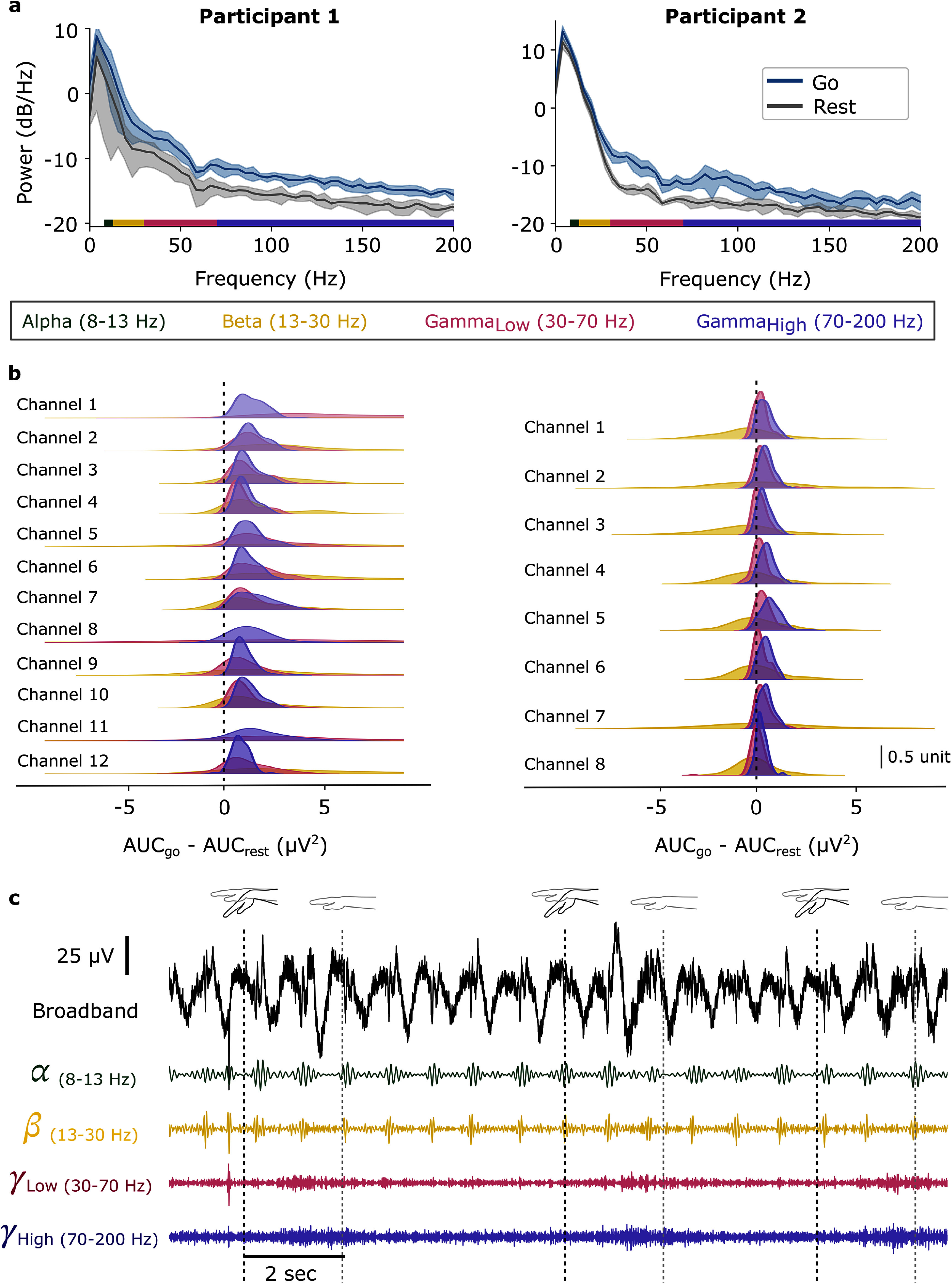
Motor signal strength analysis and preprocessing steps. (a) An example of power spectral density plots from a single channel, showing data from Participant 1 during attempted bilateral ankle movements and Participant 2 during attempted right hand movement. The gray and blue lines represent the average PSD from *n* = 10 trials recorded during rest and attempted movement epochs, respectively, with the shaded regions denoting the standard deviation. The colored bars on the *x*-axis indicate the frequency bands: alpha (8–13 Hz), beta (13–30 Hz), low gamma (30–70 Hz), and high gamma (70–200 Hz). (b) To evaluate the differences between rest and go signals, the area under the curve (AUC) was computed from the power spectral density (PSD) plots across the beta, low gamma, and high gamma frequency bands for Participant 1 and Participant 2. These differences were calculated for all channels across sessions and visualized using a kernel density plot. The vertical dashed lines indicate the *x* = 0 point, representing no difference between the rest and go conditions. (c) An example of a single-channel broadband signal filtered into specific frequency bands from Participant 2 during attempted right hand movement.

We used Welch’s PSD analysis with a window size of 256 m s and a 50% overlap to estimate the power distribution across frequencies (Parhi and Ayinala 2014, Wang *et al* 2015). The data for each channel was segmented into epochs of ‘Rest’ and ‘Go’ (i.e. attempted movement) based on the cues displayed to the participants. The PSD for each epoch was calculated and then averaged across trials separately for the rest and attempted movement states. Based on prior literature (Crone *et al* 2006, Mukamel and Fried 2012, Parvizi and Kastner 2018, Dubey and Ray 2020, Pattisapu and Ray 2023), we divided the frequency spectrum into four bands: alpha (8–13 Hz), beta (13–30 Hz), low gamma (30–70 Hz), and high gamma (70–200 Hz) (figure 2(a)),

\begin{align*}{\text{PSD}_{\text{avg}}}\left( \,f \right) = \frac{1}{T}{\text{ }}\mathop \sum \limits_{t = 1}^T \left[ {\frac{1}{{N \cdot {F_s}}}\mathop \sum \limits_{k = 1}^{{K_t}} {{\left| {{X_{kt}}\left( \,f \right)} \right|}^2}} \right]\end{align*}

where:

$\left( \,f \right)$ represents the frequency component being analyzed.

$\left( N \right)$ is the number of data points in each segment.

$\left( {{F_s}} \right)$ is the sampling frequency (2000 Hz).

$\left( {{X_{kt}}\left( \,f \right)} \right)$ is the Fourier Transform of the *k*th segment from the *t*th trial.

$\left( {{K_t}} \right)$ is the number of segments for the *t*th trial, calculated by dividing the vECoG signal into overlapping windows (window size = 256 m s) of 50% overlap.

$\left( T \right)$ is the total number of trials for a given condition (either ‘attempted movement’ or ‘rest’).

$\left( {{\text{PS}}{{\text{D}}_{{\text{avg}}}}\left( \,f \right)} \right)$ is the averaged PSD across all trials for a particular condition.

#### 2.4.2. Spectral feature extraction

Based on the PSD analysis, the difference in the area under the curve (AUC) between the rest and go phases was calculated across beta, low gamma, and high gamma frequency bands for all sessions and channels (figure 2(b)). A repeated-measures ANOVA identified significant differences among these bands, with post hoc Tukey tests confirming pairwise differences between the bands. Additionally, Wilcoxon signed-rank tests were performed to determine whether the AUC differences between rest and go phases within each band were significantly different from zero (*p* < 0.001). The low gamma and high gamma bands were subsequently isolated for further analysis using a tenth-order Butterworth zero-phase band-pass filter (figure 2(c)).

**Figure 3.**
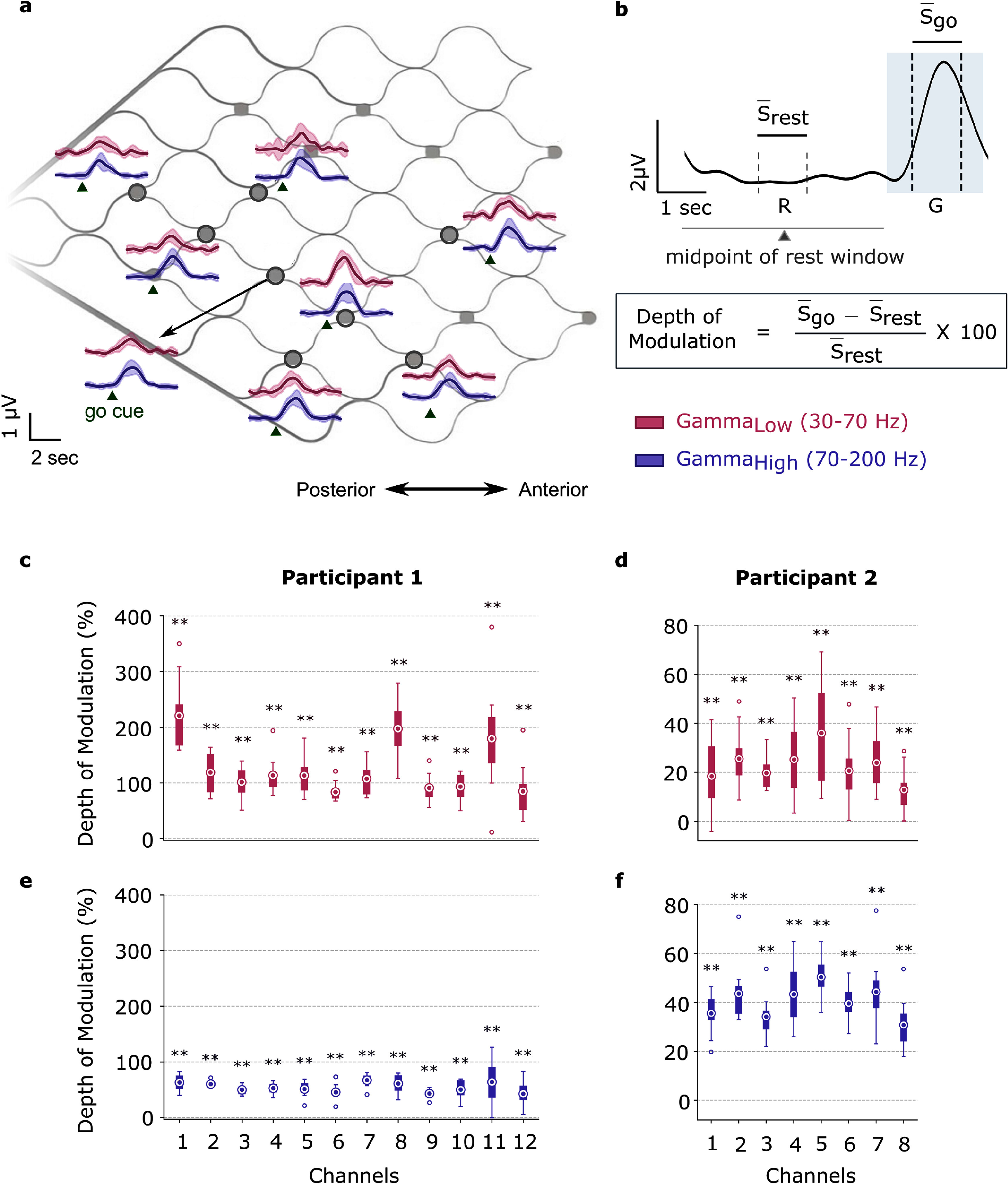
Schematic of the Stentrode with signal envelopes, depth of modulation calculation and results. (a) Schematic of the Stentrode in a flattened configuration alongside the corresponding signal envelopes recorded from Participant 2 across all electrodes during one session of right hand movement attempts. The low and high gamma signal envelopes for each electrode are indicated by solid lines representing the mean envelope taken across all trials from one session, with the shaded region indicating the standard deviation. A green triangle marks the moment the ‘go’ cue was displayed on the screen. The signals are band-pass filtered and color-coded by frequency band: pink for low gamma and purple for high gamma. (b) Depth of modulation is defined as the percentage change of average amplitude recorded during attempted movement ($\overline{S}$_go_) with respect to average amplitude of rest ($\overline{S}$_rest_). $\overline{S}$_go_ is calculated in a 1000 m s window, shifted to start 500 m s after the go cue to account for reaction time. $\overline{S}$_rest_ is calculated in a 1000 m s window around the midpoint of the rest window. (c)–(f) The depth of modulation values are shown for vECoG signals recorded as participants performed attempted movements during one session (Participant 1: both ankles; Participant 2: right hand). The analysis focuses on low gamma (a), (b) and high gamma (c), (d) features across all channels (*n* = 10 trials). For each box-plot, the embedded circle represents the mean, the bottom and top edges of the box correspond to the 25th and 75th percentiles, respectively, and the whiskers extend to the minimum and maximum values, excluding outliers. Outliers are displayed separately as unfilled circles. ** Indicates *t*-test was significant at *p* < 0.01.

We segmented the band-pass filtered signals into discrete bins of 100 m s and measured the root-mean-square (RMS) amplitude in each bin. The RMS signal was smoothed using a fifth-order lowpass filter with a cutoff frequency of 1 Hz to obtain an envelope of the vECoG signal (figure 3(a)).

#### 2.4.3. Depth of modulation (DoM) and SNR

We calculated the percentage change in the average amplitude of the vECoG signals between the rest and attempted movement states, which we refer to as the DoM. We have used DoM as a normalized measure of signal strength to compare features in the low gamma and high gamma band. Similar DoM metrics have been used by Collinger *et al* (2014), Crone (1998), Pfurtscheller and Lopes Da Silva (1999),

\begin{equation*}\text{DoM} (\%) = \left( \frac{\overline{S}_{\text{go}} - \overline{S}_{\text{rest}}}{\overline{S}_{\text{rest}}} \right) \times 100\%.\end{equation*}

where $\overline{S}$_rest_ and $\overline{S}$_go_ refer to the average of band-limited vECoG envelope signals during the rest and go states, respectively. $\overline{S}$_rest_ was calculated in the 1000 m s epoch around the midpoint of the rest preceding the ‘Go’ cue, and $\overline{S}$_go_ was computed during a 1000 m s window beginning 500 m s after the ‘Go’ cue, which allowed for the delay associated with the reaction time to the prompt (figure 3(b)). This DoM metric was calculated for each electrode on individual trials and classes of movement tested in each participant.

The same $\overline{S}$_rest_ and $\overline{S}$_go_ values were used to calculate the SNR for pairs of rest and go trials, where $\overline{S}$_rest_ represented noise and $\overline{S}$_go_ represented the signal,

\begin{equation*}\text{SNR(dB)} = 20 \log_{10} \left(\frac{\overline{S}_{\text{go}}}{\overline{S}_{\text{rest}}}\right).\end{equation*}

Linear regression was performed to analyze trends in channel-averaged SNR values across sessions over the three month period. The slope, coefficient of determination (*R*^2^), and *p*-value were calculated to assess the significance of the trend over time.

#### 2.4.4. Signal stability

We performed two types of analyses to examine the stability of vECoG signals over a 3 month testing period. For P1, we examined vECoG signals from 12 Stentrode channels recorded during the 4th, 5th, and 6th months post-implantation. For P2, we examined vECoG recordings from 8 Stentrode channels over the 8th, 9th, and 10th months post-implantation.

The first analysis focused on computing the accuracy of discriminating go-period activity from rest-period activity using an LDA classifier. The low and high gamma vECoG signal amplitudes from all channels were used to test the discriminability of rest and go signals. For each epoch of rest and go, we calculated $\overline{S}$_rest_ and $\overline{S}$_go_ respectively for every channel in both the low gamma and high gamma frequency bands. The data was pooled across sessions performed over a 3 month testing period and a 5-fold LDA classifier was used to assess offline classification accuracy. The classifier was trained on 80% data from every month and tested on 20% holdout data from every month. Additionally, we trained an LDA classifier using 80% of the first month’s data and tested it on the remaining 20% within that month, as well as on the entire datasets from the subsequent two months. This approach assessed whether the representation of motor intent learned from the initial days could generalize to decoding on future days.

The second analysis examined the stability of vECoG signals by applying principal component analysis (PCA) to aggregated multichannel signal envelopes and performing a *d*′-prime analysis to quantify the discriminability between rest and go conditions over the 3 month period. Data from all sessions was concatenated and band-pass filtered to extract multichannel signal envelopes (Wu *et al* 2023, Yesilkaya *et al* 2023). PCA was then used to reduce the dimensionality of the data, and the first principal component (PC1), which accounted for more than 60% of the total variance, was used to represent the multichannel signal envelopes as a single time series (Nurse *et al* 2018). Using the PC1 channel, we performed a *d*′-prime analysis to quantify the discriminability between rest and go conditions. For each session, the *d*′-prime value was calculated using the mean and variance of the PC1 amplitudes across all rest (noise) and go (signal) epochs within that session. The *d*′-prime formula is given as:

\begin{align*}d^{\prime} = \frac{{{\mu _{{\text{signal}}}} - {\mu _{{\text{noise}}}}}}{{\sqrt {\frac{{\sigma _{{\text{signal}}}^2 - \sigma _{{\text{noise}}}^2}}{2}} }}.\end{align*}

where *μ*_signal_ and *μ*_noise_ are the means of the PC1 amplitudes for the go and rest conditions, respectively, and *σ*^2^_signal_ and *σ*^2^_noise_ are the variances of the PC1 amplitudes for the go and rest conditions, respectively. This analysis provided a single discriminability score (*d*′) per session for each frequency band and participant, offering a longitudinal assessment of signal discriminability across the 3 month testing period. A linear regression analysis was conducted to evaluate trends in *d*′-prime values across time for P1 and P2 in the low gamma and high gamma frequency bands. The analysis included slope, *R*^2^, and *p*-value to assess statistical significance.

#### 2.4.5. Somatotopic differences

LDA was employed to examine differences in the pattern of motor activity recorded across the Stentrode during attempted movements of different limbs. The data set for P1 includes attempted movements of the right ankle, left ankle, and both ankles. The data set for P2 includes attempted movement of the right hand, left hand, and both hands. The multichannel average amplitude of rest and go measures from all trials for the low and high gamma features were pooled for LDA. A leave-one-out cross-validation (LOOCV) framework was used to evaluate the LDA model, where the model was trained on all but one sample and tested on the held-out sample. Classification accuracy was averaged across all iterations. In the LDA-transformed space, Mahalanobis distances were calculated to evaluate class separability. Within-class distances were computed by measuring the distance of each test sample from the centroid of its corresponding class in the training set. Between-class distances were determined by calculating the Mahalanobis distances between the centroids of the training classes. Both within-class and between-class distances were averaged across all iterations to quantify class consistency and distinction, respectively.

## 3. Results

We are presenting preliminary results from two participants with ALS who were implanted with a Stentrode array in the SSS adjacent to the primary motor cortex (figure 1). In this section, all values are reported as mean ± standard deviation or mean (standard deviation) for consistency.

### 3.1. vECoG detects motor activity in low and high gamma bands

The PSD plots in figure 2(a) show differences in low and high gamma band signal power for the rest and attempted movement conditions. For P1, repeated-measures ANOVA revealed significant differences in AUC differences across frequency bands (*F*(2, 2301) = 77.54, *p* < 0.001). Post-hoc Tukey tests indicated that the beta band differed significantly from both the gamma (*p* < 0.001) and high gamma bands (*p* < 0.001), while no significant difference was observed between the low gamma and high gamma bands (*p* = 0.126). Wilcoxon signed-rank tests confirmed that AUC differences for all bands (beta, low gamma, and high gamma) were significantly greater than zero (*p* < 0.001). For P2, repeated-measures ANOVA also revealed significant differences in AUC differences across frequency bands (*F*(2, 2373) = 33.04, *p* < 0.001). Post-hoc Tukey tests showed significant differences between all pairs of bands: beta and low gamma (*p* < 0.001), beta and high gamma (*p* < 0.001), and low gamma and high gamma (*p* < 0.001). Wilcoxon signed-rank tests indicated that the low gamma and high gamma bands had AUC differences significantly greater than zero (*p* < 0.001), while the beta band did not differ significantly from zero (*p* = 0.312). Both participants exhibited significant differences from zero in the low and high gamma bands, suggesting consistent modulation of these bands during task performance.

A positive DoM value indicates that the signal amplitude during the attempted movement period exceeds the amplitude during rest. For P1, the average DoM across all channels and sessions was 125.41 ± 17.52% in the low gamma band and 54.23 ± 4.52% in the high gamma band (table 1). For P2, the average DoM across all channels and sessions was 22.77 ± 4.09% in low gamma and 22.53 ± 2.04% in high gamma (table 2). In comparison, Collinger *et al* (2014) examined sensorimotor rhythm (10–40 Hz) and high gamma band (65–115 Hz) in participants undergoing epilepsy monitoring with subdural ECoG grids, reporting an average DoM of 42.7 ± 21.60% for low-frequency and 25.0 ± 11.30% for high gamma bands during action execution across participants. Examples of DoM values from one session for both the participants (P1: both ankles movement, P2: right hand movement) in the low gamma and high gamma bands are shown in figures 3(c)–(f). For P1, we show DoM examples calculated on a session 5 months post-implantation (figures 3 (c) and (e)), and for P2, we show DoM examples calculated on a session collected 10 months post-implantation (figures 3(d) and (f)). A one-sample *t*-test was performed to determine if the DoM values were significantly different from zero at *p* < 0.01. A Bonferroni multiple comparisons correction was applied. All channels exhibited DoM values significantly greater than zero for both participants.

For P1, SNR values in the low gamma band ranged from 5.06 to 9.96 dB, and in the high gamma band ranged from 2.97 to 4.44 dB (table 1). For P2, SNR values in the low gamma band ranged from 1.02 to 2.57 dB, and in the high gamma band ranged from 1.07 to 2.34 dB (table 2).

**Figure 4.**
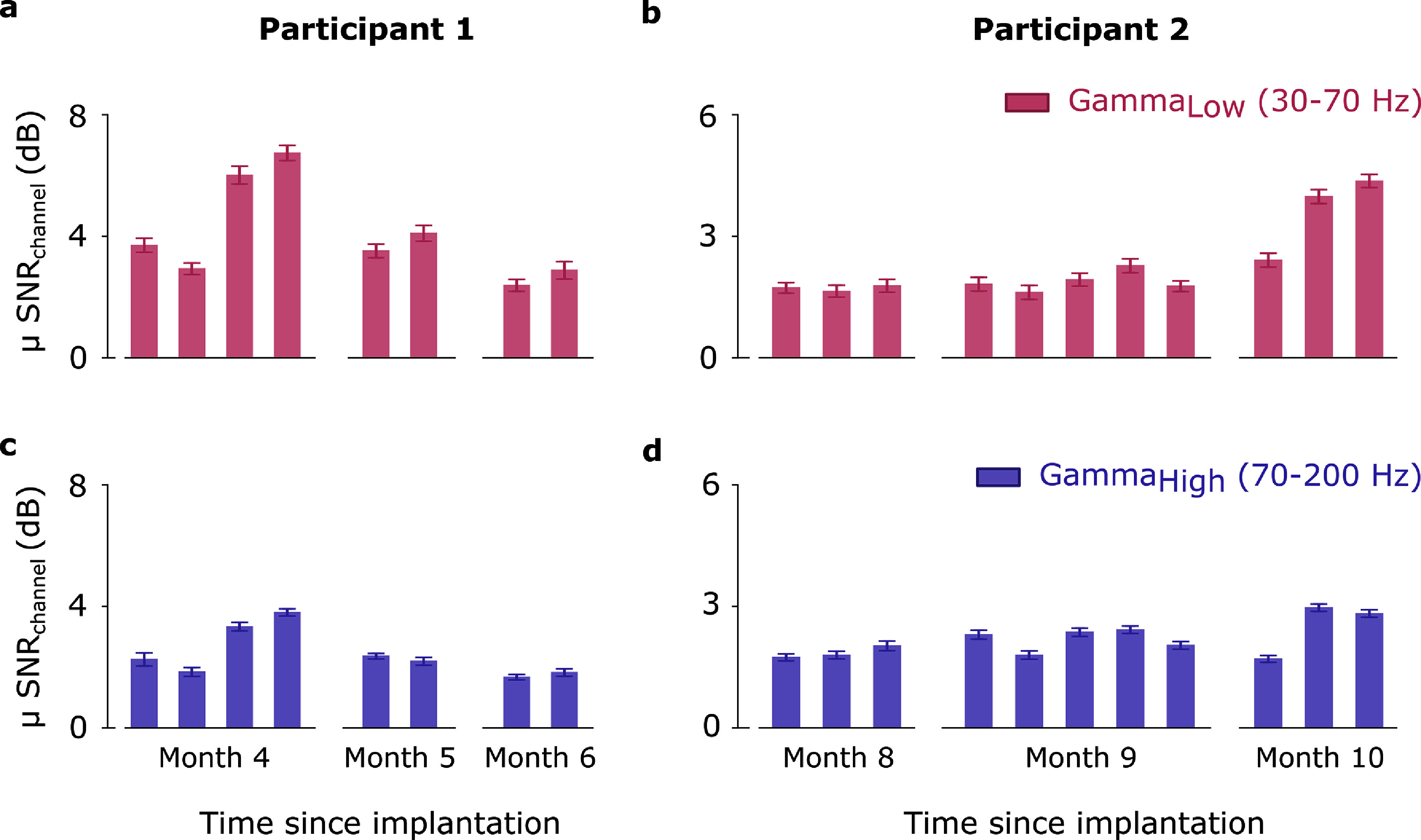
Channel-averaged signal-to-noise ratio (SNR) in the gamma and high gamma frequency bands over three months for Participant 1 and Participant 2. (a) and (c) Show the mean SNR values for Participant 1 during attempted movements of both ankles, while (b) and (d) depict the mean SNR values for Participant 2 during attempted movements of the right hand. Each bar represents the mean of all trials during a session within that month. Error bars represent the standard deviation of daily SNR values, illustrating the variability observed across days. SNR = 0 dB represents no difference between the rest and go conditions.

Figure 4 illustrates the variation in channel-averaged SNR values across three months for both participants. Each bar represents the mean of all trials during a session within that month, highlighting differences in signal quality over time and between frequency bands.

For P1, both the gamma (slope = −0.207, *R*^2^ = 0.107, *p* = 0.429) and high gamma (slope = −0.104, *R*^2^ = 0.111, *p* = 0.420) bands exhibited weak, non-significant decreasing trends, indicating stable SNR over time. For P2, the gamma frequency band showed a strong, significant increasing trend (slope = 0.227, *R*^2^ = 0.614, *p* = 0.004). Similarly, in the high gamma frequency band, a moderate, significant increasing trend was observed (slope = 0.084, *R*^2^ = 0.407, *p* = 0.035), suggesting an increase in signal strength.

### 3.2. Motor signal modulation was present over a 3 month period

**Figure 5.**
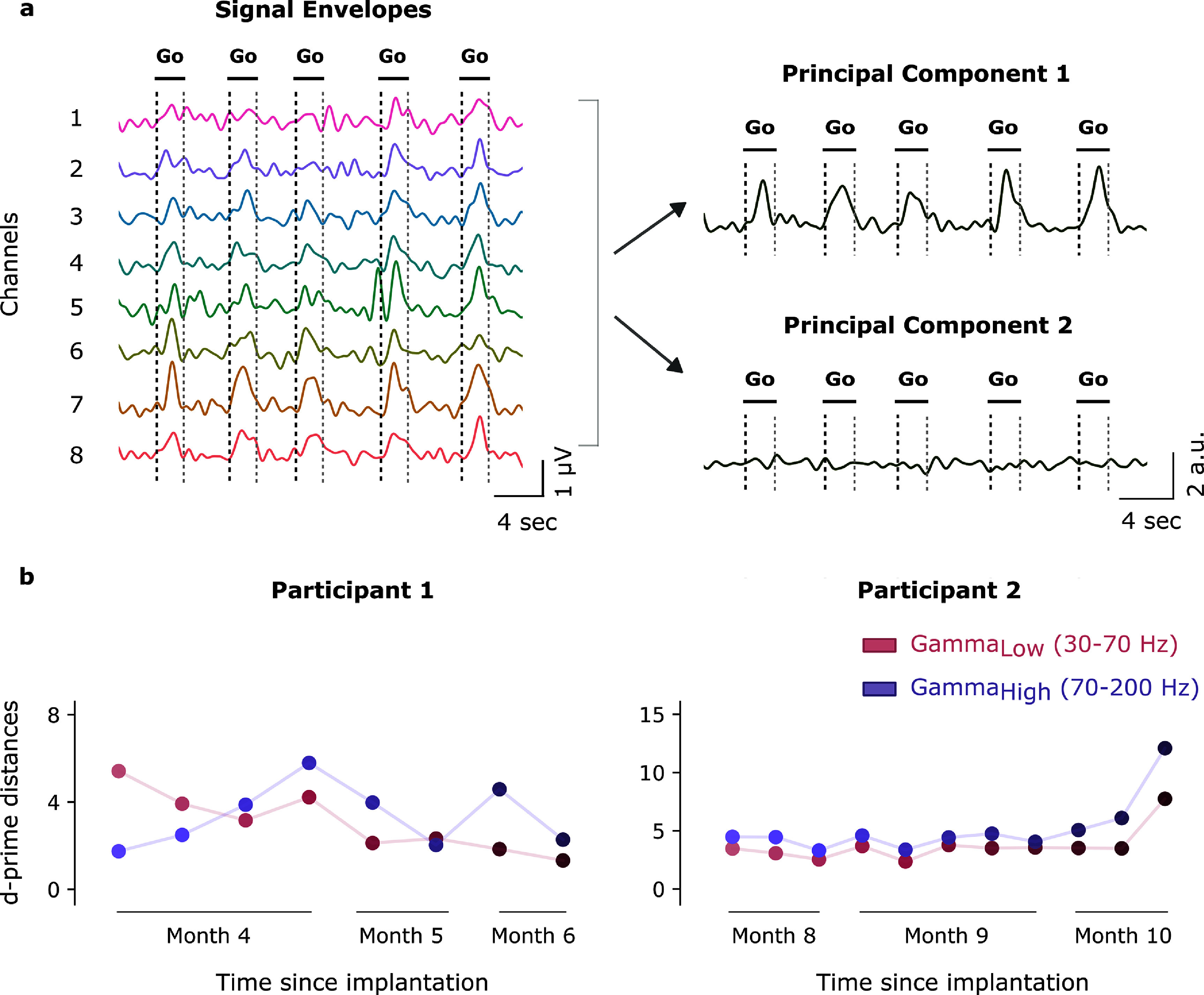
Motor signal strength over three months in the low and high gamma frequency bands during attempted movements. (a) Representative signal envelopes of low gamma frequency bands from eight channels of Participant 2 and the 1st two principal components that were derived from PCA applied to data that was pooled across 3 months of recordings. (b) The plots depict the d′-prime value for each session in low gamma and high gamma frequencies for Participant 1 and Participant 2. Each point corresponds to the *d*′-prime value calculated using the mean of rest and go values across all epochs within that session. Low gamma frequencies are shown in shades of pink, while high gamma frequencies are represented in shades of purple. Transparent pink and purple lines connect the *d*′-prime values within each frequency band to illustrate trends across sessions. Sessions from the same month are grouped by lines, and the color intensity of the points darkens progressively over time.

We examined the amplitude of motor signals in the low and high gamma bands over a 3 month testing period. The multichannel vECoG signals were consolidated using PCA. The first principal component (PC1) captured >60% of the variance in the low and high gamma vECoG signals for both participants. For P1, the fraction of variance explained by PC1 for the low gamma band was 64.3% and for the high gamma band was 72.4%. For P2, the percentage variance explained by PC1 for the low gamma band was 65.8% and for the high gamma band was 78.0 %. The large majority of variance explained by PC1 indicates a high level of covariation in the motor activity recorded across the array and underscores its utility in representing the dominant task-related features of the vECoG signals (figure 5(a)).

As was done for the single channel data, we measured the strength of the motor signal features in the composite vECoG signal by comparing the amplitude of the PC1 signals between the rest and go epochs. For P1, the data was recorded during attempted movement of both ankles beginning four months post-implantation of the Stentrode. For P2, the data was recorded during attempted movement of the right hand starting eight months post-implantation.

A Wilcoxon signed-rank test with Bonferroni correction for multiple comparisons confirmed that the low and high gamma signals were significantly larger in the go-period compared to the rest period (*p* < 0.01). It is noteworthy that for both participants and both frequency bands, the go period activity is significantly different from the rest period activity across all three months that were tested, demonstrating that the motor activity is stable over the entire duration.

The rest and go vECoG amplitude data from all channels was pooled across the 3 month testing interval. A 5-fold LDA classifier was trained for each participant in both low gamma and high gamma bands. For P1, the low gamma classifier achieved a mean accuracy of 93 ± 6%, while the high gamma classifier achieved a mean accuracy of 95 ± 2%. For P2, the low gamma classifier achieved a mean accuracy of 93 ± 3%, and the high gamma classifier achieved a mean accuracy of 96 ± 3%. The second analysis evaluated the performance of an LDA classifier trained on data from the first month and tested across the three months. The low gamma and high gamma data was combined for this classifier. For P1, the classifier achieved an accuracy of 100% in month 1, 95% in month 2, and 75% in month 3. For P2, the classifier achieved an accuracy of 95% in month 1, 97% in month 2, and 97% in month 3.

All *d*′-prime values are positive. For P1, the low gamma frequency band exhibited a significant decreasing trend (slope = −0.520, *R*^2^ = 0.836, *p* = 0.001), indicating a significant reduction in *d*′-prime values over time. In the high gamma frequency band, there was a weak, non-significant increasing trend (slope = 0.082, *R*^2^ = 0.019, *p* = 0.742). For P2, the low gamma frequency band showed a moderate increasing trend (slope = 0.244, *R*^2^ = 0.326), though the trend was not statistically significant (*p* = 0.067). Conversely, the high gamma frequency band displayed a significant increasing trend (slope = 0.456, *R*^2^ = 0.390, *p* = 0.040), reflecting a clear upward trend in *d*′-prime values over time (figure 5(b)).

### 3.3. Strongest volitional modulation was observed in both ankles for P1 and right hand for P2

**Figure 6.**
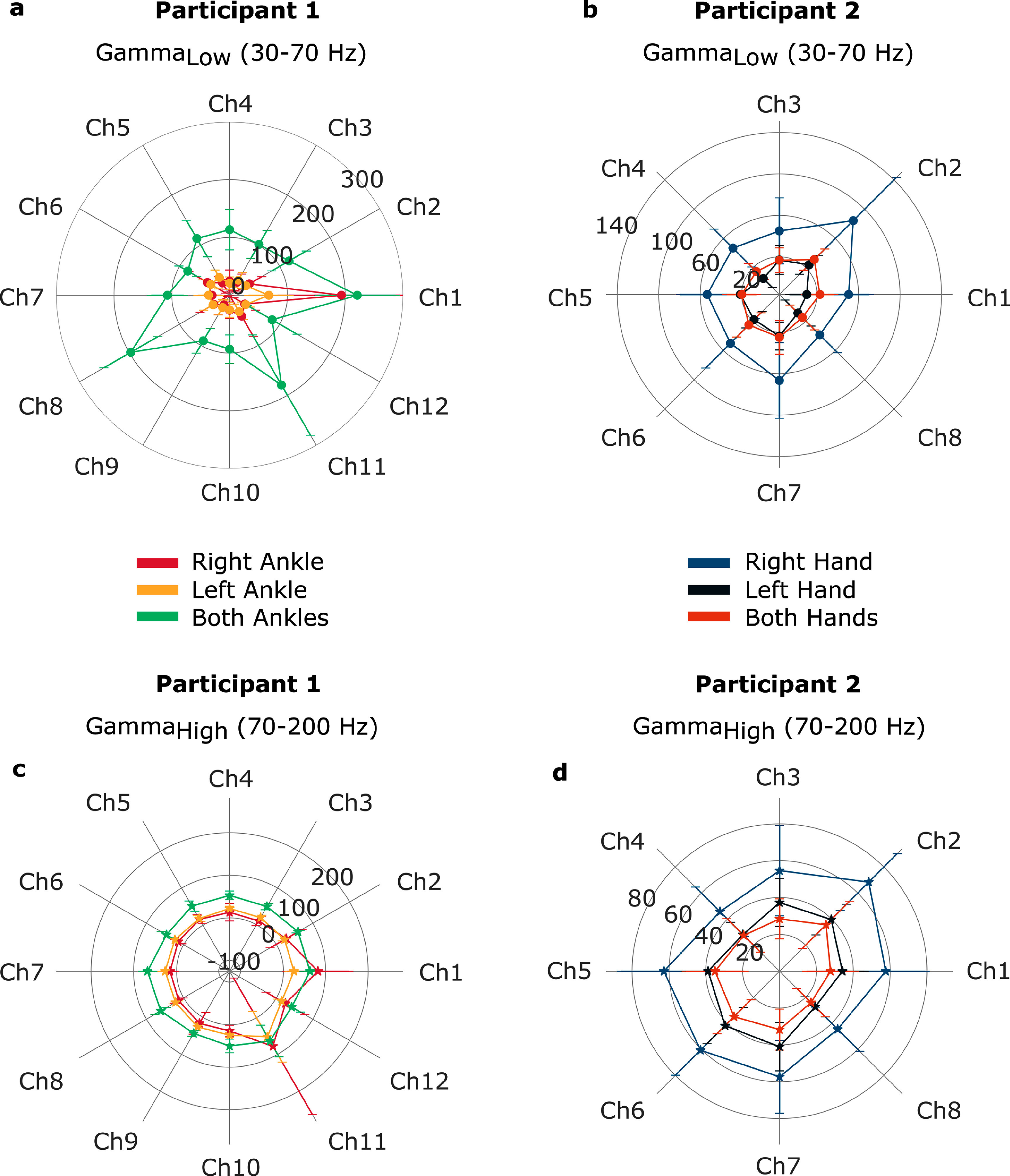
Mean and standard deviation of depth of modulation values for various attempted movements across low and high gamma frequency bands. The polar plots show the depth of modulation values for each channel as the radial distance from the center. The numbers on the radial circle represent the depth of modulation value shown by that circle. Every color represents data for a different type of attempted limb movement. Depth of modulation calculated for the low gamma frequency band is represented with circle markers and for the high gamma frequency band with star markers. (a), (c) For P1, DoM was calculated for all the channels while they performed attempted movement of right ankle, left ankle and both ankles. (b), (d) For P2, DoM was calculated for all the channels while they performed attempted movement of right hand, left hand and both hands.

To compare vECoG signals associated with different limb movements, we calculated the DoM values for all channels in both the low gamma and high gamma bands while participants were cued to perform attempted movements of various limbs. For P1, we analyzed vECoG signals recorded during attempted movement of the right ankle, left ankle, and both ankles. For P2, we compared vECoG signals during attempted movement of the right hand, left hand, and both hands. For each movement, the mean and standard deviation of the DoM values across trials were computed for each channel (figure 6). For P1, we analyzed data recorded 4–5 months post-implantation and for P2 we analyzed data recorded three months post-implantation.

We calculated the average and standard deviation DoM values taken across channels for each type of attempted movement. For P1, the average DoM values for attempted movement of both ankles, right ankle, and left ankle in the low gamma frequency band were 125.4 ± 44.8%, 44.3 ± 45.5%, and 34.1 ± 11.3%, respectively. The channel-averaged DoM values for the high gamma band signals for attempted movement of both ankles, right ankle, and left ankle were 54.2 ± 8.2%, 28.0 ± 24.2%, and 24.6 ± 8.9%, respectively. For P2, these values for the attempted movement of the right hand, left hand and both hands in the low gamma frequency band were 54.5 ± 13.6%, 16.1 ± 6.7%, and 21.4 ± 5.4%, respectively. The attempted movement of the right hand, left hand and both hands in the high gamma frequency band was 54.5 ± 7.6%, 36.0 ± 5.3%, and 30.6 ± 4.0%, respectively.

We further evaluated the discriminability of motor signals produced during different types of attempted movements using LDA. The average amplitude of the low and high gamma signals measured during the *rest* ($\overline{S}$_rest_) and *go* ($\overline{S}$_go_) epochs (see figure 3(b)) for each channel were combined prior to applying LDA.

**Figure 7.**
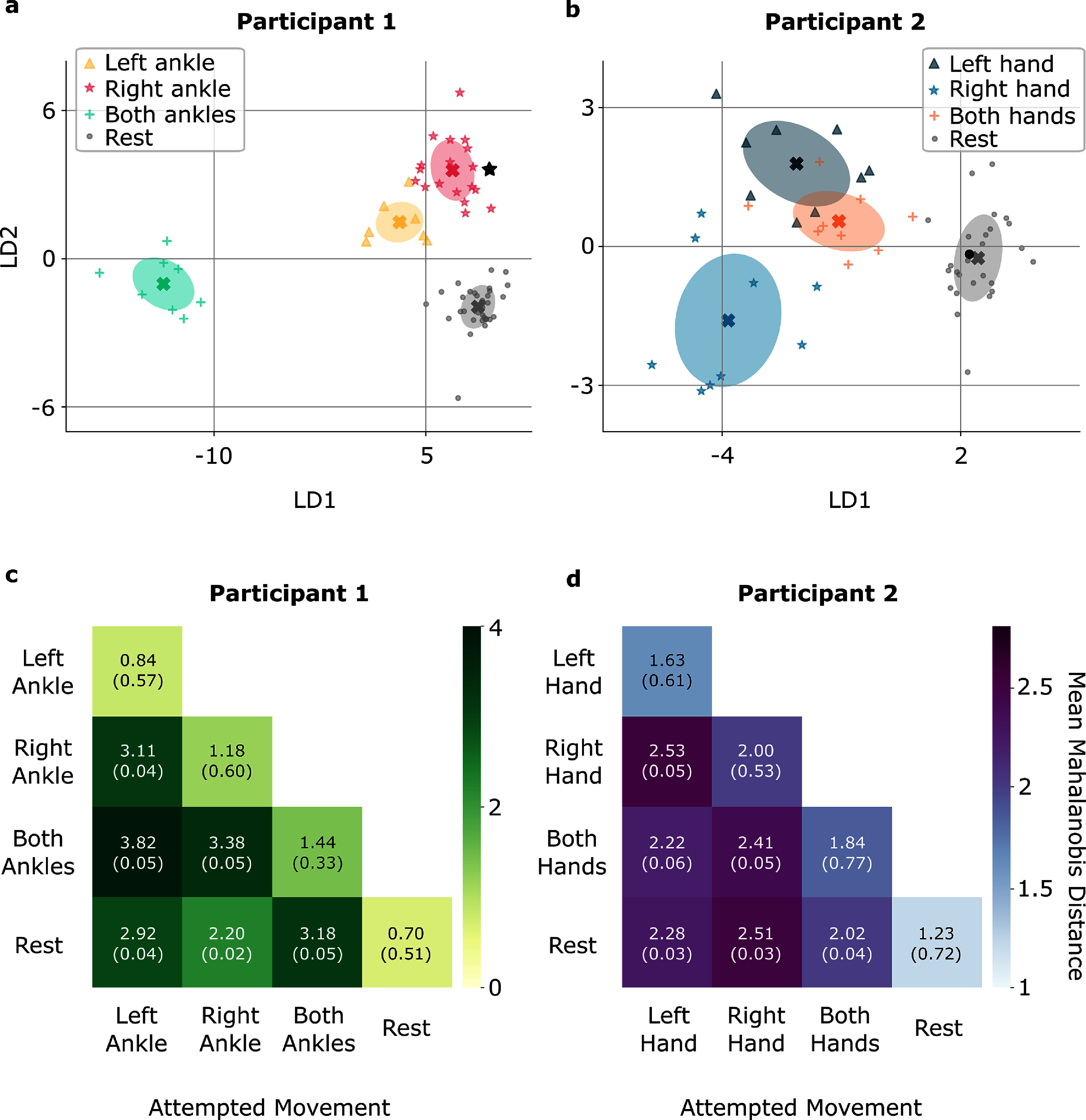
Linear discriminant analysis of combined low and high gamma vECoG features for limb movement classification and inter-class separability. (a), (b) The combined low and high gamma features are projected into a two-dimensional Linear Discriminant (LD) space, displaying results from one iteration of the leave-one-out cross-validation as an example. The plot illustrates the separation between attempted movement and rest conditions within the LD space. Training data are represented by shapes specific to each class and colored differently for clarity, while the test point, correctly classified, is marked in black with the same shape as the training data of its respective class. Ellipses illustrate the variability and spread of each class within the LD space, while centroids, marked with crosses, represent the mean position of each class, both derived from the training data. (c), (d) The matrices show the mean Mahalanobis distances and their standard deviations in the LDA-transformed space, averaged across iterations. The diagonal elements represent the within-class distances, indicating the dispersion of test data points around their respective training centroids. The off-diagonal elements show the between-class distances, reflecting the separability between different movement and rest conditions for that participant.

Figures 7(a) and (b) shows the projections of the labeled vECoG amplitude features into the LD space for one iteration of the LOOCV, including the test point. In figures 7(a) and (b) the ellipses represent the variability and spread of each class in the two-dimensional LDA space, based on the covariance of the training data points within each class. The size and orientation of the ellipses are determined by the eigenvalues and eigenvectors of the covariance matrix, providing insight into the distribution and overlap of the classes. The centroids, marked with crosses, indicate the mean position of each class in the LDA space. The test point for each iteration is shown in black, with the shape matching that of the corresponding class.

For P1, the overall classification accuracy achieved through LOOCV was 91.43%. The class-wise accuracy was 87.50% for the left ankle condition, 78.95% for the right ankle condition, 100.00% for the both ankles condition, and 97.14% for the rest condition. Within-class Mahalanobis distances showed the smallest dispersion for the rest condition (0.70 ± 0.51) and the largest for the both ankles condition (1.44 ± 0.33). Between-class Mahalanobis distances highlighted that the both ankles condition demonstrated the greatest separability with all three other conditions, including the left ankle (3.82 ± 0.05), the right ankle (3.38 ± 0.05), and the rest condition (3.18 ± 0.05) (figure 7(c)).

For P2, the overall classification accuracy was 70.37%. The class-wise accuracy was 44.44% for the left hand condition, 55.56% for the right hand condition, 44.44% for the both hands condition, and 92.59% for the rest condition. Within-class Mahalanobis distances showed the smallest dispersion for the rest condition (1.23 ± 0.72) and the largest for the right hand condition (2.00 ± 0.53). The right hand condition exhibited the highest separability from all three other conditions, including the left hand (2.53 ± 0.05), the both hands condition (2.41 ± 0.05), and the rest condition (2.51 ± 0.03) (figure 7(d)).

Due to the limited dataset with all three movements recorded at timepoints in close proximity, this preliminary analysis may introduce potential bias in the discriminability results. This analysis provides an initial understanding of the separation between different classes. Further optimization and the use of larger datasets are expected to improve the separability, leading to a more accurate representation of multiclass classification.

## 4. Discussion

The present study aimed to measure the strength and stability of motor signals in the low gamma and high gamma bands of vECoG signals recorded with Stentrodes implanted endovascularly in the SSS of two participants with severe paralysis due to ALS. We quantified motor modulation by measuring amplitude changes in the low and high gamma band signals between sequential epochs of rest and attempted movement. These signals were also analyzed for stability over a period of three months. The results of this preliminary study highlight the Stentrode’s ability to reliably detect volitional motor-related neural signals offering a significant promise for the future of high-quality endovascular BCI recordings without the need for craniotomies.

The results of this study demonstrate that the Stentrode can effectively differentiate between the rest and attempted movements through changes in the amplitude of vECoG signals across both the low gamma and high gamma frequency bands. The previous study by Mitchell *et al* (2023) identified modulation within the beta frequency band to be effective for distinguishing motor intent from rest in human participants implanted with a Stentrode. Their analysis was conducted on data from the SWITCH trial (NCT03834857), whereas our study focuses on participants from the COMMAND EFS. A key methodological difference lies in the referencing system. Mitchell *et al* (2023) utilized a fixed reference anchored to the IRTU in the chest, while our study employed an updated system with a switchable reference. Specifically, we chose a local reference to minimize ECG artifacts, which anecdotally reduced beta-band signal modulation. This difference in referencing methodology likely accounts for the observed lower separability in the beta band for distinguishing motor intent from rest in our analysis. In contrast to Mitchell *et al* (2023), our findings demonstrate greater separability in the low and high gamma bands for distinguishing motor intent from rest.

These findings are consistent with previous studies that used ECoG grids implanted surgically on the surface of the brain. Previous research on ECoG-based BCIs also observed an increase in power in the low gamma and high gamma bands during motor imagery tasks (Crone 1998, Miller *et al* 2007, Chestek *et al* 2013, Blakely *et al* 2014, Branco *et al* 2017, Freudenburg *et al* 2019). John *et al* (2018) reported SNR values in sheep models, which were not expressed in dB. To enable comparison with our study, these values were converted to dB using 20 × log_10_ (reported SNR). It is important to note that the SNR values in John *et al* (2018) were derived from electrically evoked responses resulting from median nerve stimulation in sheep. The SNR for Stentrode recordings in sheep exhibited a unimodal distribution with an interquartile range of 1.71 (4.6 dB), aligning with comparable SNR values reported for epidural recordings (Oxley *et al* 2016, John *et al* 2018). In our study, the mean SNR values were 6.75 ± 0.37 dB and 3.69 ± 0.28 dB for low and high gamma, respectively, in P1, and 1.72 ± 0.25 dB and 1.73 ± 0.13 dB for low and high gamma, respectively, in P2.

This highlights that the Stentrode’s placement within the SSS provides high-quality signal capture, potentially matching the performance observed previously with ECoG (John *et al* 2018), and maintains this quality for up to 10 months post-implantation. For P1, the DoM and SNR is higher in the low gamma band compared to the high gamma band. In contrast, for P2, the DoM is higher in the high gamma band, but the SNR is comparable in both bands. This variation could be attributed to the different movements being analyzed for each participant. The significant increase in attempted movement amplitude compared to rest as shown by the high DoM values in these bands suggests strong cortical representation of motor intent that the Stentrode can capture despite its novel, less invasive placement.

Previous studies of the Stentrode in sheep model have demonstrated that the array and leads become integrated into the vessel wall, likely resulting in higher device stability and increased proximity to the motor cortex (Oxley *et al* 2016, Opie *et al* 2017). We also demonstrate the stability in signal differentiation up to six months and 10 months from the date of device implantation for P1 and P2 respectively. Our analysis over three months demonstrates the presence of motor modulation in both participants throughout the study period. While *d*′-prime values showed trends such as a decrease in the low gamma band for P1 and an increase in the high gamma band for P2, all values remained positive, indicating the motor-related neural activity was distinguishable from rest. Similarly, the evaluation of an LDA classifier trained on combined low and high gamma data confirms that motor modulation signals were sufficient to achieve notable classification accuracy across three months, despite some variability between participants. This suggests the potential long-term use while maintaining signal quality, a common challenge in many BCI systems (Ryapolova-Webb *et al* 2014, Downey *et al* 2018).

We examined the signal strength of low and high gamma signals across different types of attempted limb movements in the low gamma and high gamma frequency bands. Motor activity levels varied across the array for each movement type. The overall classification accuracy for decoding multiple attempted movements was 91.43% for P1 and 70.37% for P2. Considering that the Stentrode is positioned along the brain’s midline, above the region of activation corresponding to lower limb signals according to the motor homunculus (Kocak *et al* 2009, Roux *et al* 2020), we expected to observe the strongest signals during attempted movement of the ankles. This was consistent with our finding for P1 where we observed the highest DoM values for the attempted movement of both ankles. Additionally, the both ankles and rest conditions demonstrated strong separability and high classification accuracy. Even though the representation of the upper limbs is more lateral to the ankles, we observe high values of DoM for attempted movement of the right hand in P2. While classification accuracy was similar across hand movements, the right hand condition exhibited the highest separability from other classes in Mahalanobis distance. This might be attributed to the intuitiveness of the movement for the participant or the Stentrode’s proximity to the supplementary motor area (SMA). Studies show that the area of SMA for forelimb representation lies next to the hindlimb representation of M1 (Mitz and Wise 1987). Additionally, the findings by Willett *et al* (2020) suggest that mixed selectivity in the motor cortex may also contribute to this phenomenon. Their work highlights how movements across different body parts can be represented in overlapping cortical areas, suggesting a more flexible and compositional neural coding scheme that may explain the decodability of hand movements from the brain’s midline.

In this study, we measured the amplitude of motor activity by measuring the difference in the average amplitude of signals taken within 1000 m s windows of the rest and attempted movement periods to accommodate variations in the timing of neural activity. However, since the motor activity associated with attempted movement is transient, measuring the average amplitude over a large window results in an underestimate of the DoM and SNR. Consequently, the actual strength of motor activity relative to rest may be significantly higher.

## 5. Limitations

This preliminary efficacy study offers insights into the potential of the Stentrode to detect motor signals in low and high gamma bands in people with severe paralysis due to ALS. However, we acknowledge some important limitations of the work presented here.

The study population includes only two people and both are males with severe paralysis due to ALS. It will be important to extend this investigation to a larger and more diverse population of people with paralysis who may benefit from BCI. Furthermore, the analysis of motor signaling was conducted offline, and the neural signals may behave differently during real-time BCI operation. Real-time analysis of BCI control is essential to fully assess the operational capabilities of the Stentrode in everyday use.

The analysis of chronic stability was limited to a 3 month span in each participant. While the results indicate that motor signals were discriminable throughout this period, it will be helpful to examine the vECoG signals over a larger timespan to ensure that the BCI can function reliably and effectively for potentially several years. On this point, it is also important to consider the potential loss of motor neurons and cognitive decline that may occur due to disease progression. ALS is a neurodegenerative disease, and its impact on neural signal characteristics over time is not fully understood. Multiple factors can affect signal quality, including the proximity of the device to the motor neurons and the participant’s level of consciousness, which can fluctuate due to the disorder. Moreover, previous studies have shown that 15%–30% of people are unable to modulate their brain signals to operate BCIs (Becker *et al* 2022, Kim *et al* 2023). Thus, there are multiple potential failure modes and we are currently limited in our ability to identify or mitigate them.

## 6. Conclusion

This preliminary study was conducted on the data from the first two participants of the COMMAND EFS. It demonstrates that volitional motor-related neural signals can be detected accurately in two participants with severe ALS-induced paralysis. Significant increases in vECoG signal amplitudes during attempted movements were observed, indicating strong cortical representation of motor intent. This remained consistent over a 3 month period. Binary classification of motor intent was performed accurately over a 3 month testing interval, demonstrating the potential for the Stentrode to support simple BCI tasks such as binary switch control. Furthermore, the high overall classification accuracy of 91.43% observed for P1 across different types of attempted movements indicates strong class separability, highlighting the potential for achieving binary control of multiple output channels. For P2, an overall accuracy of 70.37% was achieved, suggesting variability in class separability. A larger dataset is needed to better evaluate the feasibility and consistency of such control across a broader population. The long-term stability of signal detection over several months highlights the Stentrode’s potential for durable BCI solutions in individuals with severe paralysis.

## Data Availability

The data cannot be made publicly available upon publication because they contain commercially sensitive information. The data that support the findings of this study are available upon reasonable request from the authors.
